# Editorial: Application of deep learning in biomedical image processing

**DOI:** 10.3389/fmolb.2026.1816401

**Published:** 2026-03-20

**Authors:** Zhanlin Ji, Li Zhao, Ivan Ganchev

**Affiliations:** 1 College of Mathematics and Computer Science, Zhejiang A&F University, Hangzhou, China; 2 Beijing National Research Center for Information Science and Technology, Institute for Precision Medicine, Tsinghua University, Beijing, China; 3 TRC/ECE, University of Limerick, Limerick, Ireland; 4 Faculty of Mathematics and Informatics, University of Plovdiv “Paisii Hilendarski”, Plovdiv, Bulgaria; 5 Informational Modeling Department, Institute of Mathematics and Informatics − Bulgarian Academy of Sciences, Sofia, Bulgaria

**Keywords:** artificial intelligence (AI), biomedical imaging, deep learning (DL), image classification, image segmentation, object detection

In the era of data-driven medicine, biomedical imaging has evolved from a purely diagnostic tool to a cornerstone of precision healthcare. The confluence of deep learning (DL) and biomedical image processing has catalyzed a paradigm shift, enabling unprecedented performance in image segmentation, disease detection, and predictive analysis. Biomedical imaging datasets are inherently complex, characterized by high dimensionality, variability in acquisition protocols, and necessity for clinically actionable insights. DL models, with their ability to automatically extract hierarchical features, have emerged as a transformative solution to these challenges. From enhancing the precision of laryngeal surgery to accelerating the screening of infectious diseases, the articles collected in the Research Topic “Application of Deep Learning in Biomedical Image Processing” underscore the translational impact of artificial intelligence (AI)-driven imaging research and showcase cutting-edge original research that exemplifies the breadth and depth of DL applications in biomedical imaging, expanding the boundaries of what is possible in this dynamic field.

An automatic laryngoscopic image segmentation system (Zhang et al.) addresses a critical clinical need by extending the Segment Anything Model (SAM) for laryngoscopic analysis. By optimizing prompt engineering, the proposed system achieves robust glottis annotation and vocal fold segmentation, supporting real-time guidance during laryngeal procedures and advancing the standard of care for voice disorders. Experimental evaluation demonstrates that the system achieves superior segmentation performance, comparable to that of supervised models. The study also introduces useful metrics extracted from the vocal folds’ masks that cannot be derived from the glottis masks alone.

LarynxFormer (Mæstad et al.), a framework for processing and segmenting laryngeal images, introduces a dedicated transformer-based architecture, designed to handle the anatomical complexity of laryngeal tissues. The framework demonstrates superior performance in segmenting vocal folds and detecting pathological lesions, offering a scalable solution for both clinical practice and research. The authors conclude that models, collaboratively trained on distributed datasets, could facilitate the development of more robust and generalizable frameworks.

Hybrid feature fusion in cervical cancer cytology (Niu et al.) presents a dual-module framework for automated cervical cytology analysis that integrates radiomics, DL, and reproducibility validation. By fusing handcrafted radiomic features with DL embeddings, the framework delivers state-of-the-art performance in cervical cancer lesion detection and classification, with explicit attention to cross-site reproducibility—a critical factor for clinical deployment.

Research on the application of a multi-model cascaded DL framework in pathological diagnosis of osteosarcoma (Yao et al.) introduces a cascaded DL pipeline that integrates multi-modal pathological imaging data. The proposed framework automates the detection of osteosarcoma subtypes, reducing inter-observer variability and accelerating diagnostic workflows in musculoskeletal oncology. Evaluation results reveal excellent performance of the framework, indicating its high potential for future clinical application in osteosarcoma management.

Skin disease diagnosis using decision and feature level fusion of deep features (Uddin et al.) leverages a multi-model fusion strategy to integrate outputs from convolutional neural networks (CNNs) and vision transformers. By combining feature-level and decision-level fusion, the proposed framework achieves high accuracy in classifying a diverse range of skin lesions, providing a scalable tool for tele-dermatology. A suggestion is made for the utilization of lightweight ensemble models to address practical challenges in resource-constrained real-time clinical settings.

Using novel DL models for rapid and efficient assistance in monkeypox screening from skin images (Deng et al.) addresses the global health challenge w.r.t. monkeypox disease spreading by employing self-attention mechanisms, feature pyramid integration, and attentional strategies to amalgamate image features across varying scales. The proposed model enables rapid, high-throughput screening of skin lesions, supporting timely outbreak response, reducing the burden on diagnostic laboratories, and lowering the workload of medical professionals.

LT-YOLO (Li et al.), a long-term YOLO-based model for stenosis detection on invasive coronary angiography, extends the YOLO architecture with temporal attention mechanisms to analyze sequential angiographic frames. The proposed model improves the detection of coronary artery stenosis by capturing dynamic blood flow patterns, thus enhancing the precision of interventional cardiology procedures. Extensive experiments on invasive coronary angiography (ICA) video sequences demonstrate the model’s superior performance over state-of-the-art models, particularly in detecting previously-underexplored small stenoses (<50%).

A DL-based and radiomics-driven algorithm for automated identification of May-Thurner syndrome (MTS) in Iliac computed tomography venography (CTV) imaging (Chen et al.) combines DL segmentation with radiomic feature analysis to identify MTS in CTV scans. The algorithm automates the detection of venous compression, enabling early intervention and reducing the risk of thromboembolism. The obtained results suggest the potential clinical utility of the developed algorithm for MTS diagnosing, offering a non-invasive and efficient alternative to traditional methods.

RetinalVasNet (Yao Z. et al.) is a DL method for robust retinal microvasculature detection, utilizing a specialized CNN architecture for retinal vessel segmentation. The proposed method outperforms existing methods in handling image artifacts and low-contrast vessels, enabling early detection of diabetic retinopathy and glaucoma through microvascular analysis. The optical coherence tomography (OCT) seems a promising future application of RetinalVasNet.

DL-based algorithms, utilized for magnetic resonance images (MRI)-based acute and subacute ischaemic stroke lesion segmentation (Baaklini et al.), are systematically reviewed and assessed, based on their performance, along with providing supplementary meta-analysis and pilot evaluation of key results. Multiple model artefacts are compared, discussing their potential impact on segmentation performance. The use of a U-Net configuration with residual connections seems the most appropriate one for performing such segmentation task.

The 10 articles presented in this Research Topic demonstrate that DL is no longer a theoretical concept but a practical tool driving clinical innovation. However, significant challenges remain ahead, including the need for larger, more diverse datasets, explainable AI models to build clinical trust, and regulatory frameworks to ensure safe deployment. As the field advances, interdisciplinary collaboration between researchers, clinicians, and data scientists will be essential to translating these breakthroughs into routine patient care.

The presented Research Topic not only celebrates the current achievements of DL in biomedical image processing but also charts a course for future research. The collected articles provide a roadmap for developing more accurate, efficient, and accessible AI-driven imaging tools that will redefine the future of medicine. We extend our gratitude to the authors, reviewers, and editorial team for their contributions to this collective effort.

